# A New Planar Microwave Sensor for Building Materials Complex Permittivity Characterization

**DOI:** 10.3390/s20216328

**Published:** 2020-11-06

**Authors:** João G. D. Oliveira, José G. Duarte Junior, Erica N. M. G. Pinto, Valdemir P. Silva Neto, Adaildo G. D’Assunção

**Affiliations:** 1 Department of Communication Engineering, Federal University of Rio Grande do Norte, Caixa Postal 1655, Natal CEP 59078-970, RN, Brazil; gjoao187@gmail.com (J.G.D.O.); garibs@ufrn.edu.br (J.G.D.J.); vpraxedes.neto@gmail.com (V.P.S.N.); 2 SIDIA Institute of Science and Technology, Manaus CEP 69055-035, AM, Brazil; 3 Avenida Universitária Leto Fernandes, Federal Rural University of the Semi-Arid, Caraúbas CEP 59780-000, RN, Brazil; erica.gurgel@ufersa.edu.br

**Keywords:** microwave sensor, microstrip antenna, log-periodic, high directivity, concrete characterization, dielectric measurements

## Abstract

A new microwave sensor is proposed to characterize the complex relative permittivity of building non-magnetic materials and used in the characterization of three concrete samples. The proposed sensor structure consists of a log-periodic planar antenna with microstrip elements tilted forward by an angle β and printed, alternately, on the top and bottom sides of a dielectric layer. The operation principle is based on the measurement of the scattering parameters S_11_ and S_21_ in a free space propagation transmitter-receiver setup, for both cases with the material under test (MUT) sample (non-line-of-sight, NLOS) and without it (line-of-sight, LOS). A prototype is fabricated and measured to determine the scattering parameters of concrete samples. After measurements, the obtained results are used in the efficient and accurate Nicolson–Ross–Weir (NRW) method, making it possible to estimate the values of the complex relative permittivity of the concrete blocks. The sensor design is demonstrated from initial simulations to measurements for validation of the developed prototype. The obtained results for the complex relative permittivity of concrete are in agreement with those available in the literature and the difference between the simulated and measurement results for the sensor antenna resonant frequency is 4.71%. The used measurement setup can be applied to characterize different types of solid or liquid dielectric materials.

## 1. Introduction

In the current scenario of mobile communications technologies, several studies have been carried out seeking to discover and minimize possible problems that may arise in the communication channels. Several studies are being carried out showing that elements positioned between the transmitter/receiver terminals severely affect the characteristics and the transmission capacity of any radio link [1,2,3,4,5]. In a communication system, one of the most common types of obstruction to the propagation of electromagnetic waves happens in the presence of materials used in the construction of buildings. In addition, the most used building material is concrete, being chosen for several works due to its ease of manufacture, low cost, and durability. Therefore, the study of the electrical characteristics of this material has become increasingly important, in view of the numerous possible scenarios in mobile communications. As presented in [6], it is possible to monitor the hydration process of the concrete depending on the chloride variation by measuring the permittivity, making it possible to confirm these properties by comparing them with other microstructural characterization techniques, such as energy dispersive X-ray (EDX) and scanning electron microscopy (SEM) [7]. It is worthwhile to mention the effectiveness of microwave sensors for the characterization of concrete properties and other construction materials, without destroying its matrix [8,9,10,11,12]. These sensors can be used in works already carried out and thus identify problems without damaging the existing concrete or mortar structure.

Other types of sensors were developed, such as the microwave double waveguide sensors, which serve to identify small gaps between the concrete structures and the metal [13,14], enabling an evaluation of the size of the gap between the concrete and metal, and from there technical decision-making regarding the maintenance of the structures. Small gaps or even the appearance of cracks, variation in the mechanical properties, and the density of the material can also be monitored using non-invasive techniques [15,16,17,18]. This process can be applied to assess the change in the properties of concrete structures and their degradation over time or even after changes in climatic conditions or natural phenomena.

Compressive strength is one of the most studied properties in concrete. From it, the amount of load that the concrete supports and its durability are determined. However, a characteristic that affects this material is its water/cement ratio. Thus, producing a microwave sensor that can measure electrical properties [18,19,20,21,22,23] and relate them to the amount of water and mechanical properties is very promising.

Identifying the properties of the concrete is clearly important but, in some structures, it is necessary to identify the reinforcement materials (fibers) that were used to produce the concrete. Therefore, the study of a microsensor based on a V-shaped resonator (VSR) was proposed as an economical and non-destructive solution to detect the type and percentage of content in fiber-reinforced concrete [24]. In [25], a time domain technique is presented for determining the real and imaginary parts of the permittivity and permeability of samples of linear materials in the frequency domain.

In the last decades, a great interest has been observed from researchers worldwide in the determination of the electromagnetic properties of materials for applications in wireless communication systems. In [26], a step-by-step procedure is proposed for the characterization of the permittivity of dielectric materials using the NRW method. In [27], non-resonant measurement methods are presented and used to determine the permittivity of dielectric materials. In [28,29,30], methodologies are proposed for the electromagnetic characterization of concrete based on radar models relating the losses of the propagating wave. In [31,32,33,34], studies were carried out on the electrical and magnetic parameters of concrete-based materials.

In addition, one of the most used methods in the electromagnetic characterization of materials, including concrete, is the Nicolson–Ross–Weir (NRW) method [35,36,37]. This method is based on the characteristics of propagation and reflection of an electromagnetic wave within a set of transmitter/propagation medium and sample/receiver. Parameters, such as the wavelength and scattering coefficients (S11 and S21), are used to obtain the electrical and magnetic characteristics of the propagation medium or of a material under test (MUT) sample.

In this context, this work proposes a new microwave sensor to be used in the characterization of the complex relative permittivity of concrete samples, based on simulated and measured values of the attenuation constant, for the propagation of electromagnetic waves. The used technique can be considered as non-destructive, as the physical structures of the samples were not changed. Some properties of the electromagnetic wave propagation and its interaction with lossy dielectric media are presented. Agreement is observed between the simulated and measured results.

## 2. Materials and Methods

### 2.1. Electrical Properties of Non-Magnetic Materials

The electromagnetic waves that propagate through buildings interact with their physical structures, causing a loss of propagation that depends on the electrical characteristics of the materials used in their construction. Therefore, a key factor in the study of wave propagation between different media is the identification of each medium through which the wave propagates. Basically, the medium identification is defined by its main electrical parameters, which are the electrical permittivity, ε, electrical conductivity, *σ*, and magnetic permeability, *μ*. For building materials, a medium can be identified as conductor if *σ* ≫ *ωε* and dielectric if σ ≪ *ωε* [38], where ω is the angular frequency.

The behavior of an electromagnetic wave propagating in the $z^{+}$ direction is determined from the solution of the wave equation, being represented by the expressions of the electric and magnetic fields indicated in Equations (1) and (2) [39]:

(1) $$E\left(t,z\right)=\;E_{0}e^{-j\omega t-\gamma z},$$

(2) $$H\left(t,z\right)=\;H_{0}e^{-j\omega t-\gamma z}=\frac{E_{0}}{\eta }e^{-j\omega t-\gamma z},$$

where *γ* is the propagation constant, given by *γ* = *α* + *jβ*, where *α* is the attenuation constant and *β* is the phase constant, and *η* is the intrinsic impedance of the medium given by $\sqrt{\mu /\varepsilon }$. Therefore, it is easy to understand how the electrical properties of materials are great impact factors on the behavior of waves that come to interact with their structures.

### 2.2. Method for Relative Permittivity Determination Using Scattering Parameters

The most widely used method is that of Nicolson–Ross–Weir (NRW) [40], which enables the values of the complex relative permittivity ($\varepsilon_{r}=\;\varepsilon_{r}^{\prime }\;-j\varepsilon_{r}^{\prime \prime }$) and complex relative permeability ($\mu_{r}=\;\mu_{r}^{\prime }\;-j\mu_{r}^{\prime \prime }$) to be obtained simultaneously. For non-magnetic materials, $\mu_{r}=1$. The NRW method requires, in the measurement setup, information on some parameters, such as the material thickness, d, and the distance between the material under test (MUT) and the antennas, L.

**Figure 1:**
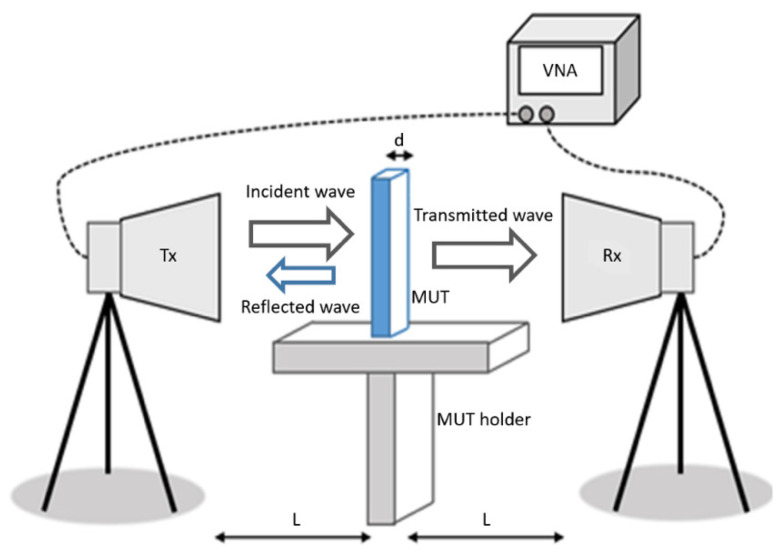
Illustration of the measurement setup.

Figure 1 shows the schematic diagram of the measurement setup for wave transmission/reception to be used in the measurement of the MUT scattering parameters. Basically, the measurement setup is composed of a vector network analyzer (VNA), two horn antennas, and accessories. Measurements are carried out in free space. The required dimensions L and d are indicated.

As shown in Figure 1, part of the EM wave incident on the MUT sample will be reflected to port #1 and measured by the reflection coefficient (S11), and part will pass through it and be transmitted to port #2, being measured by the transmission coefficient (S21). Additionally, a small part of the EM wave incident on the MUT sample will be refracted and transmitted with lag to port #2, affecting the reflection coefficient (S21) measurement, unless microwave absorbers are used to minimize this effect. Then, the measured scattering parameters (S11 and S21), the thickness of the MUT sample (d), and its distance to the Tx and Rx antennas (L) are used in the expressions of the NRW method [41] to determine the electrical characteristics of the MUT sample.

The main equations of the NRW method to determine the relative permittivity and magnetic permeability are shown in Equations (3) to (5) [26,42]:

(3) $$\mu_{r}=\frac{1+\Gamma }{\Lambda \left(1-\Gamma \right)\sqrt{\frac{1}{\lambda_{0}^{2}}-\frac{1}{\lambda_{c}^{2}}}},$$

(4) $$\varepsilon_{r}=\frac{\lambda_{0}^{2}}{\mu_{r}}\left(\frac{1}{\lambda_{c}^{2}}+\frac{1}{\Lambda }\right),$$

(5) $$\frac{1}{\Lambda }=-{\left[\frac{1}{2\pi L}ln\left(\frac{1}{T}\right)\right]}^{2},$$

where $\lambda_{0}$ and $\lambda_{c}$ are the wavelengths in free space and at the cutoff frequency, respectively.

The transmission, *T*, and reflection, Γ, coefficients of the MUT sample are determined using the measured scattering parameters (S11 and S12) and Equations (6) to (8), presented in [26,42]:

(6) $$K=\frac{(S_{11}{}^{2}-S_{21}{}^{2})+1}{2(S_{21)}},$$

(7) $$\Gamma =K\;\pm \sqrt{K^{2}-1},\;\;\;\;\left|\Gamma \right|\leq 1,$$

(8) $$T=\;\frac{(S_{11}-S_{21})+\Gamma }{1-\left(S_{11}+S_{21}\right)\Gamma }.$$

In this work, the propagation channel was modelled as a two-port network (quadripole) using an equivalent transmission line and the scattering matrix, [S]. Thus, for non-magnetic materials, the expression for the relative permittivity, $\varepsilon_{r},$ is obtained as shown in Equation (9) [27]:

(9) $$\varepsilon_{r}={\left[\left(j\frac{c}{\omega L}\right)\ln\left(T\right)\right]}^{2},$$

where *c* is the free space velocity, and ω is the angular frequency.

The scattering parameters S11 and S21 are represented in phasor form, with the module and phase indications, or as complex numbers, with the indications of the real and imaginary parts. Therefore, the transmission, T, and reflection, Γ, coefficients of the MUT sample (Equations (6) to (8)) can also be expressed as phasors or complex numbers. Consequently, the use of complex numbers in Equations (3) to (5) results in obtaining the real and imaginary parts of the electrical permittivity and magnetic permeability. For non-magnetic materials, as in the case of most building materials, the relative magnetic permeability, $\mu_{r}$, is equal to 1.

### 2.3. Description of the Concrete Used

Concrete is any product or mass produced from the use of a cementitious field [43]. It can be produced from a mixture of hydraulic cement, water, gravel aggregate, fine aggregate, and chemical additives. The mixture of these materials forms a plastic paste, and from the cement hydration reactions, the concrete hardens until it creates a rock-like consistency. With this, we can classify concrete as a composite material.

Composite materials have the characteristic of obtaining properties that materials alone cannot. In concrete structures, properties, such as durability, mechanical resistance, and porosity, are the most researched, usually through destructive tests. Therefore, the electrical characterization of this material becomes interesting due to the possibility that it is not necessary to extract a sample from the structure and still obtain data that are related to other properties of the concrete.

In this work, the proposal was to research a concrete conventionally used in Brazil, with a 1: 2: 3 ratio and mechanical strength of 25 MPa, composed only of Portland cement, granite gravel, quartz sand, and water. In addition, it is known that the concrete hydration process and the amount of water present in its structure directly influence the properties of the concrete. Therefore, after studying the curing of the material and realizing that after 180 days of curing, the concrete already had a hydration process close to 100%, it was decided to use samples with more than 180 days of curing, stored in a place sheltered from the weather and without the presence of water.

## 3. Proposed Antenna Sensor Design

**Figure 2:**
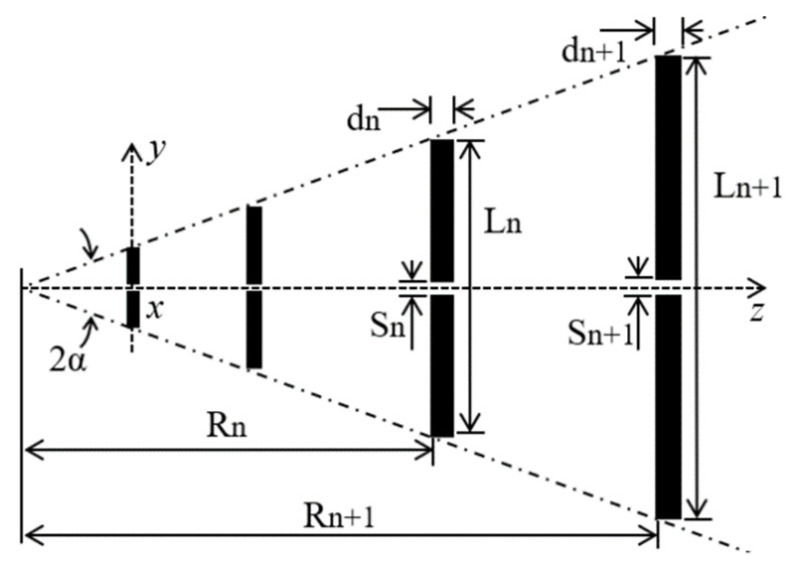
Classical geometry of a wire log-periodic antenna.

The development of a new planar antenna sensor is proposed for the electrical characterization of concrete material. The chosen characterization technique is efficient and non-destructive, requiring measurements of the propagation of an electromagnetic wave in air, between the terminals of two antennas, in the cases without and with concrete material blocks. In view of these requirements, a proposed microstrip antenna sensor was designed to achieve a directional radiation pattern, acceptable directivity, small size, low weight, low cost, and easy fabrication. Its structure is inspired by the log-periodic antenna geometry shown in Figure 2 [44].

Here, *N* is the number of dipoles of the antenna array and the wire dipoles dimensions are lengths, *L_n_*_+1_, diameters, *d_n_*_+1_, spacings, *S_n_*_+1_, and positions, *R_n_*_+1_, for *n* = 0, 1, 2,…, *N* − 1. The angle 2α defines the imaginary envelope where the length limits of the dipole elements are contained.

In the design of a wire log-periodic antenna, its dimensions are defined by the geometric ratio, *τ*, and spacing factor, *σ*, according to the expressions shown in Equations (10) and (13), respectively, which are related to the antenna directivity, number of dipoles, *N*, designed bandwidth, *B_s_*, and angle α, as shown in Equations (12) and (13):

(10) $$\frac{1}{\tau }=\frac{L_{n+1}}{L_{n}}=\frac{S_{n+1}}{S_{n}}=\frac{d_{n+1}}{d_{n}}=\frac{R_{n+1}}{R_{n}},$$

(11) $$\sigma =\frac{R_{n+1}-\;R_{n}}{2L_{n+1}},$$

(12) N=1+ln(Bs)ln(1τ),

(13) $$\alpha =\tan^{-1}\left[\frac{1-\tau }{4\sigma }\right].$$

**Figure 3:**
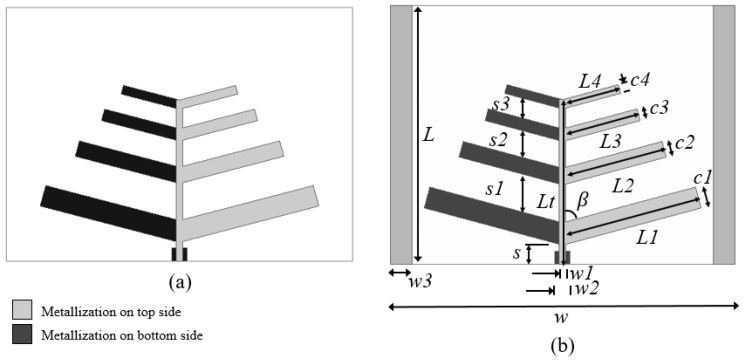
Straight connected log-periodic microstrip structures. (**a**) Antenna. (**b**) Proposed antenna sensor with two laterally placed wide strip parasitic elements.

In the microstrip antenna design, inspired by the log-periodic antenna geometry, narrow strip dipoles, tilted forward by an angle *β*, are printed on both sides of a dielectric FR4 substrate layer, as shown in Figure 3. The arms of the dipoles placed at the right side of the antenna are interconnected and printed on the top side of the dielectric substrate and those of the left side are interconnected and printed on the bottom side. The proposed log-periodic antenna dimensions are determined, using Equations (10) and (11), as a function of the operating frequency, *f*, scale factor, *τ*, and relative spacing, σ. In addition, a small number of narrow strip dipoles, *N* = 4, was chosen to get a small-sized antenna.

Two log-periodic microstrip antenna geometries were proposed and analyzed, one without (Figure 3a) and one with (Figure 3b) parasitic elements. The only difference between these antenna geometries is the use of two symmetric and laterally placed wide strips on the top side of the antenna substrate layer in Figure 3b.

To make the design and manufacture of the proposed log-periodic microstrip antenna easy, it was fed through the straight connection of the strip dipole arms placed at the right and left side of the antenna (Figure 3), which were printed on the top and bottom sides of the dielectric substrate, respectively.

The proposed antenna sensor geometry, shown in Figure 3b, was simulated and analyzed using Ansys HFSS software. Parametric analyses were carried out focusing on the antenna sensor resonant frequencies, reflection coefficient, radiation pattern, and gain performances. The structural parameters of the proposed antenna sensor are given in Table 1 and a summary of the main antenna parameters results is presented in Table 2.

A prototype of the proposed antenna sensor was fabricated using microstrip technology on a FR-4 dielectric substrate, with a relative permittivity of 4.4, loss tangent of 0.02, and thickness of 1.57 mm. Thereafter, measured results were obtained for the frequency response of the antenna sensor reflection coefficient and return loss parameters.

**Figure 4:**
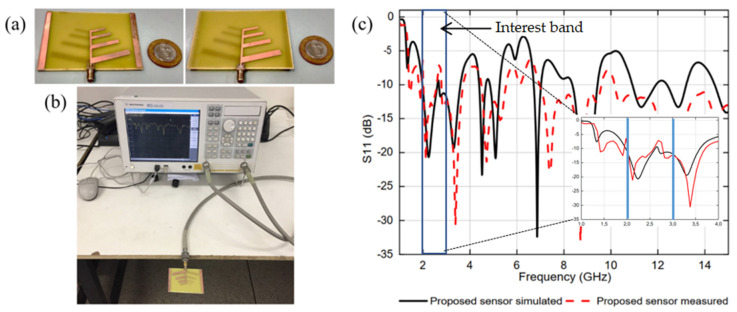
Photos of the (**a**) proposed antenna sensor prototype, top view (left side) and bottom view (right side), and (**b**) measurement setup. (**c**) Reflection coefficient simulation and measurement frequency responses.

Figure 4 shows photos of the antenna sensor prototype and measurement setup, as well as simulation and measurement results of the reflection coefficient, S11 (dB). Good agreement is observed between the simulated and measured results with multiple operation bands at 2.25, 3.32, 4.56, 5.22, 7.43, 8.73, and 12.9 GHz. In this work, the resonance band at 2.25 GHz was chosen for the antenna sensor application.

Moreover, resonance bands related to the electrical lengths of the dipoles and the effective permittivity of the used dielectric substrate (FR4), neglecting the electromagnetic coupling between the array elements, are obtained at 1.41 GHz (dipole-L1), 1.88 GHz (dipole-L2), 2.31 (dipole-L3), and 3.30 (dipole-L4). Other resonance bands are due to the electromagnetic coupling between the antenna sensor strip dipole elements.

In the performed simulation, the antennas were positioned in the x-y plane, being oriented in the positive direction of the y axis, from the longest to the shortest elements. The results of the simulated reflection coefficients for the two antennas in Figure 3 are similar but that of the antenna with two conducting strip parasitic elements (Figure 3b) presented a better resonance condition in the frequency range between 2 and 3 GHz, the frequency range of interest.

Figure 4 shows photographs of the proposed antenna sensor prototype (Figure 4a) and measurement setup (Figure 4b). Figure 4c shows the reflection coefficient simulation and measurement frequency responses. The frequency range of interest is indicated as the sensing range. The choice of the resonance band at 2.25 GHz was related to the vector network analyzer (VNA) availability and to the growing number of communication system applications in the lower microwave band (2–6 GHz).

**Figure 5:**
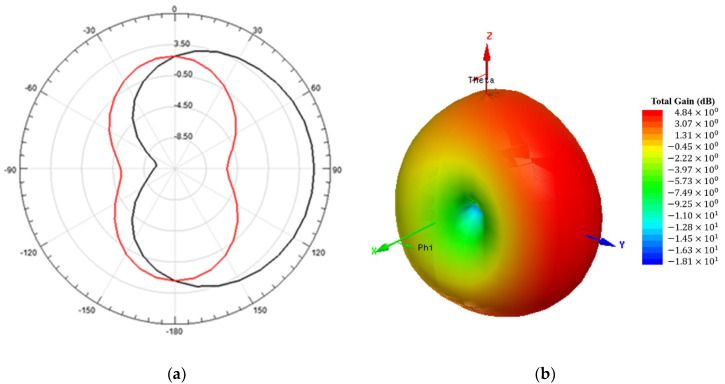
Simulated radiation pattern of the proposed antenna sensor at 2.25 GHz. (**a**) 2-D radiation pattern at ϕ = 0° (x-z plane), in red color, and ϕ = 90° (y-z plane), in black color. (**b**) 3-D radiation pattern, with gain total (dB) results.

Figure 5 shows the simulated 2-D and 3-D radiation pattern results of the antenna sensor, shown in Figure 3b, at 2.25 GHz.

The radiation patterns of the two antennas shown in Figure 3 are similar, with maximum radiation in the positive direction of the y axis and minimum radiation in the x axis direction, somewhat resembling an omnidirectional radiation pattern shifted in the positive direction of the y axis.

## 4. Experimental Procedure and Results

**Figure 6:**
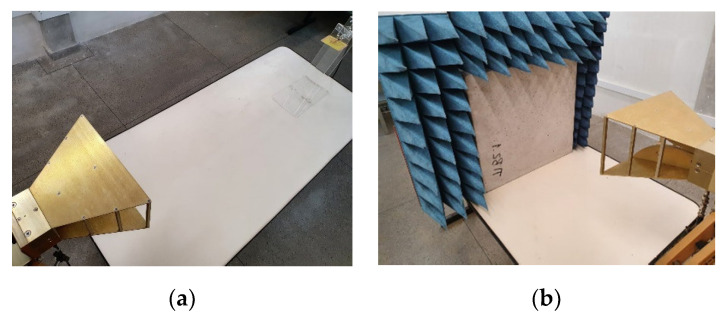
Photos of the measurement setup, showing the proposed antenna sensor, horn antenna, absorbers, and concrete block: (**a**) Setup without the MUT sample; (**b**) Setup with the MUT sample of concrete.

**Figure 7:**
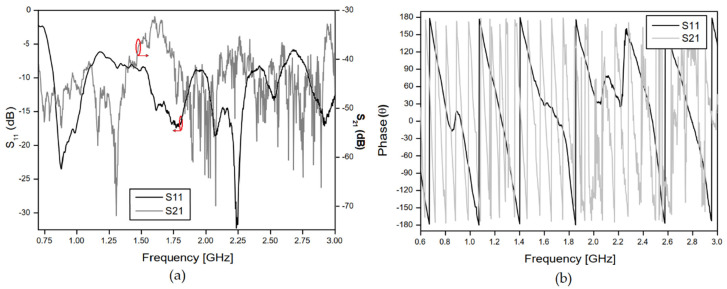
Reflection (S11) and transmission (S21) coefficients’ measured results for the free space measurement setup: (**a**) Magnitude; (**b**) Phase in degrees.

The measurement setup was basically constituted by the proposed antenna sensor, used as a transmitter, one horn antenna, with an operating range from 700 MHz to 12 GHz, a VNA connected to the antennas and microwave absorbers, which enabled to obtain measured results of the reflection and transmission coefficients for two cases: without and with insertion of an MUT sample. Figure 6a shows the measurement setup of the case without any MUT sample placed between the sensor and the horn antenna. Figure 6b shows the setup measurement with the insertion of the concrete MUT sample. In the measurement setup, reflection and scattering effects were minimized using microwave absorbers. The measured results for the free space S11 and S21 parameters are shown in Figure 7.

**Figure 8:**
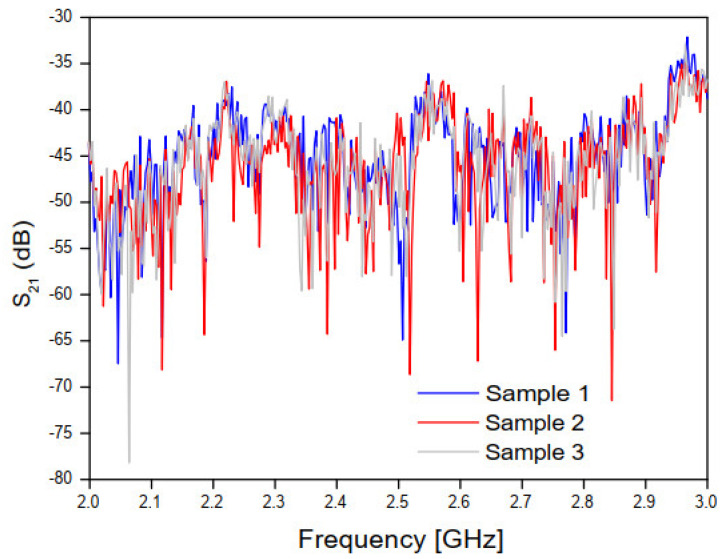
Measured results of the magnitude of the transmission coefficient for the three concrete block samples.

**Figure 9:**
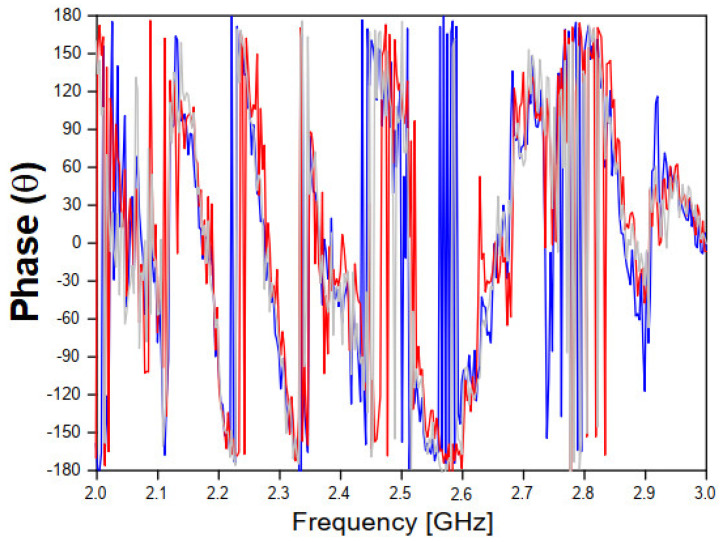
Measured results of the phase of the transmission coefficient for the three concrete block samples.

As shown in Figure 6a,b, the antennas were placed at a distance of 80 cm and the concrete block was located in the center position. Figure 8 and Figure 9 shows that the insertion of the concrete block, with a thickness of 5 cm, between the transmitter/receiver terminals, has a great effect on the phase of the received signal (S21 parameter). This is an expected result due to the scattering phenomenon and to the different properties of the concrete material sample, including its relative permittivity (which is different from the free space one), through which the EM wave should propagate. These results were used to characterize the MUT sample material that was inserted between the sensor and the receiving antenna. In the experimental procedure, measurements were carried out for four concrete blocks, and the obtained results of the reflection (S11) and transmission (S21) coefficients, including magnitudes and phases, were saved for further processing.

Figure 8 and Figure 9 show, respectively, the measured results of the transmission coefficient magnitude and phase (in degrees) for the four samples of concrete.

As shown in Figure 8 and Figure 9, a great similarity is observed between the measured results for the transmission coefficient (magnitude and phase) frequency behavior, due to the good uniformity of the four concrete blocks samples, which will help to get accurate analysis results.

### Results of The NRW Method

The results of the measurement carried out without and with the insertion of three concrete blocks placed between the terminals of the transmitting and receiving antennas were used, according to the NRW method, to (indirectly) determine the value of the complex relative permittivity of the concrete. The investigated concrete material is considered a non-magnetic material. Therefore, the relative magnetic permeability, *μ*_r_, is 1.

**Figure 10:**
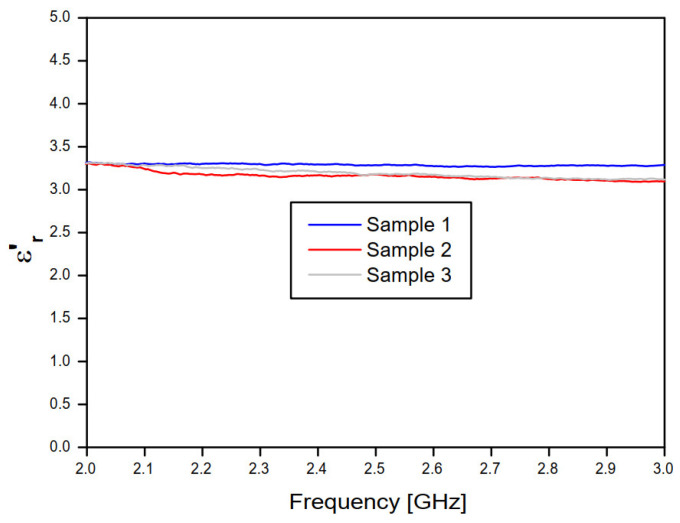
Real part of the relative permittivity of the three concrete samples, according to the NRW method and the complex transmission coefficient (S21) measured results.

**Figure 11:**
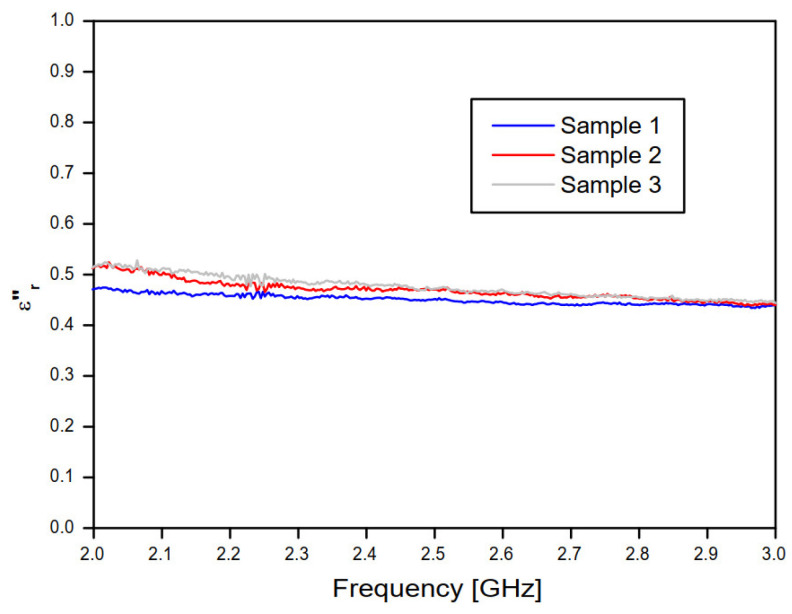
Imaginary part of the relative permittivity of the three concrete samples, according to the NRW method and the complex transmission coefficient (S21) measured results.

Figure 10 and Figure 11 show, respectively, the real and imaginary parts of the relative permittivity of the concrete material samples, as a function of frequency, according to the NRW method and the measured values of the magnitude and phase of the transmission coefficient (S21) of the concrete material blocks (Figure 8 and Figure 9).

According to Figure 10, the frequency behavior of the real part of the relative permittivity values, at lower frequencies (0.75 to 1.5 GHz) and for concrete samples 2 and 3, an excellent agreement is observed, despite the low deviation observed for the concrete sample 4 results. Nevertheless, at this frequency range, high deviation results are observed for concrete sample 1 results. Additionally, at higher frequencies (1.5 to 3 GHz) and for concrete samples 2, 3, and 4, excellent agreement is observed, despite the low deviation observed in the sample 1 results. In Figure 10, the frequency behavior curves of the imaginary relative permittivity converged to $\varepsilon_{r}^{\prime }$ ≊ 3.39.

Therefore, the used measurement setup is very appropriate to the considered higher frequency range and this result is related to the positioning distance of the concrete block and the distance between the antenna sensor and horn antenna, with respect to the operating frequencies.

In Figure 11, the frequency behavior curves of the imaginary part of the relative permittivity converged to $\varepsilon_{r}^{\prime \prime }\;$ ≊ 0.37.

**Figure 12:**
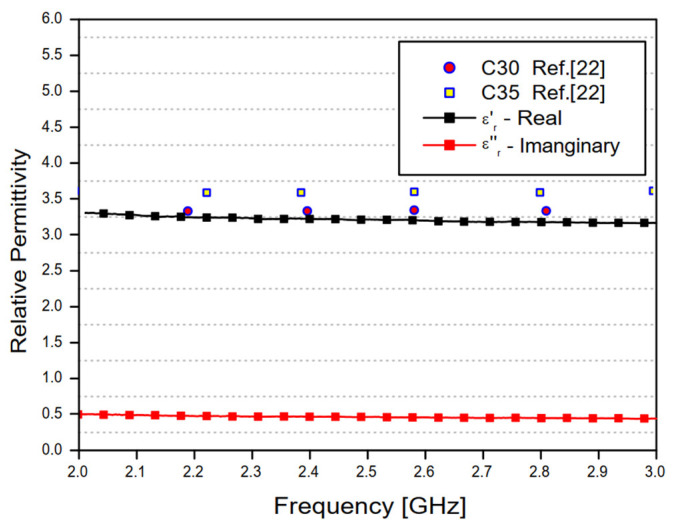
Average values of the real and imaginary parts of the concrete material relative permittivity obtained in this work and the real relative permittivity of two different samples measured in [22].

Figure 12 shows the average values of the real and imaginary parts of the concrete material relative permittivity shown in Figure 10 and Figure 11, as well as values of the real part of the relative permittivity of two different samples measured in [22].

As shown in Figure 10 and Figure 11, the relative permittivity values depend on the operating frequency. However, estimated values can be obtained through the average calculation within a specific frequency range. In this work, average values for the concrete material relative permittivity were calculated in the frequency range from 2 to 3 GHz, resulting in the complex relative permittivity value *ε_r_* = 3.39 − *j*0.37. The main results obtained in this analysis are shown in Table 3 and compared to other results in the available literature.

As shown in Table 3, the value of the real part of the relative permittivity, $\varepsilon_{r}^{\prime }$, of the concrete varies between 3.02 and 4.94, according to the available literature, being fundamentally dependent on the percentage of water in the mixture used in the block manufacture and the number of days of curing. In addition, the concrete value of $\varepsilon_{r}^{\prime }$ decreases as the number of days between the date of manufacture and that of measurement increases, up to approximately 28 days. Table 4 shows the results obtained in this work for the complex relative permittivity of concrete.

## 5. Conclusions

A new planar microwave sensor composed of a log-periodic antenna with tilted forward dipoles was developed to characterize the relative permittivity of concrete blocks. The used technique is based on the measurement in free space without and with the presence of concrete blocks samples, placed between the antenna sensor (Tx) and the horn antenna (Rx) terminals. Then, the measured results of the transmission coefficient (magnitude and phase), S21, were used according to the NRW method to estimate the concrete material complex relative permittivity.

Considering the main characteristics of the proposed sensor, such as a low profile, low cost, small size, ease of manufacture, and acceptable directivity values, it has great potential and can be used to characterize different types of materials. After the measurements of three concrete samples, it was possible to extract the approximate value of the complex relative permittivity of the measured concrete.

The results obtained show that, after a long curing period, the values of the complex permittivity of concrete blocks present little or no influence from the percentage of water in their composition, since the values found in the literature on the relative permittivity of liquids are higher than 50 and the values found in the measurement are lower than 5.

The main contributions of this work are related to the development and application of a new planar sensor with acceptable directivity and a wide bandwidth. In addition, an accurate analysis is enabled on the determination of the electrical characteristics of dielectric materials, as these parameters are directly dependent on the frequency at which they are estimated. In addition, the use of the proposed sensor significantly reduces the complexity and cost of the setup measurement, besides being used in a nondestructive technique.

As a proposal for future works, it would be interesting to investigate the application of the proposed sensor in the nondestructive characterization of other solid materials, as well as the development of other types of planar sensors.

## Figures and Tables

**Table 1:** Structural parameters of the proposed antenna sensor.

Parameters	Descriptions	Values
*w* (mm)	Total sensor width	80
*L* (mm)	Total sensor length	60
*w*1 (mm)	Top feed line width	1.5
*w*2 (mm)	Bottom feed line width	3.5
*w*3 (mm)	Laterally placed wide strip width	5
*L*1 (mm)	Dipole arm length 1	32.5
*L*2 (mm)	Dipole arm length 2	24.12
*L*3 (mm)	Dipole arm length 3	17.85
*L*4 (mm)	Dipole arm length 4	13.16
*Lt* (mm)	Center line length	38.08
*c*1 (mm)	Dipole width 1	5
*c*2 (mm)	Dipole width 2	3.74
*c*3 (mm)	Dipole width 3	2.8
*c*4 (mm)	Dipole width 4	2.09
*S* (mm)	Spacing between feed point and dipole 1	4.61
*s*1 (mm)	Spacing between dipoles 1 and 2	8.77
*s*2 (mm)	Spacing between dipoles 2 and 3	6.19
*s*3 (mm)	Spacing between dipoles 3 and 4	4.63
*β* (deg)	Angle of tilted forward dipoles	75

**Table 2:** Simulated results for the proposed antenna sensor main parameters.

Parameters	Values
Maximum directivity	3.53
Maximum gain	3.47
Radiation efficiency	98.25%
Front to back ratio	33.18

**Table 3:** Concrete material relative permittivity values available in the literature.

Reference	$\varepsilon_{r}^{\prime }$	$\;\varepsilon_{r}^{\prime \prime }$
[22] C30	3.66	-
[22] C35	3.87	-
[28]	4.94	0.69
[29] C4	3.64	0.08
[29] A4	3.89	0.10
[30] 0.2%	4.61	0.18
[31] 0.2%	3.02	-
[31] 0.3%	3.25	-
[32] 0.2%	4.60	0.18
[33] 17–18	4.47	-
[34] WC 0.2	4.39	0.27
This work	3.39	0.37

**Table 4:** Obtained results for the complex relative permittivity of concrete.

Parameter	Mean	Standard Deviation
$\varepsilon_{r}^{\prime }$	3.39	0.2438
$\varepsilon_{r}^{\prime \prime }$	0.37	0.0935
